# Comparison of measured and predicted energy density of an oral care chew for dogs

**DOI:** 10.1017/jns.2017.24

**Published:** 2017-06-15

**Authors:** Danielle Nuttall, Richard Butterwick, Katja Strauhs, Phil McGenity

**Affiliations:** 1 Mars Care and Treats, Birstall WF17 9LU, UK; 2 WALTHAM Centre for Pet Nutrition, Waltham-on-the-Wolds LE14 4RT, UK; 3 Mars Petcare Germany, Eitzer Straße 215, D-27283 Verden (Aller), Germany

**Keywords:** Metabolisable energy, Canine treats, Digestible energy, Predicted *v.* analysed energy content, C&T, care and treat, DE, digestible energy, GE, gross energy, ME, metabolisable energy, NFE, N-free extract, NRC, National Research Council, PME, predicted metabolisable energy

## Abstract

The dog chew studied here is a starch-based, twin-screw cooker extruded dog care and treat (C&T) product with oral health benefits. The manufacturing process and nutrient profile of such products are markedly different from those of main meal pet foods. Predicted metabolisable energy (PME) in pet food is calculated using equations derived from main meal feeding studies so it is unclear whether these equations can be applied to C&T products. The present study aimed to directly measure metabolisable energy (ME) content of the dog chew in dogs and compare with calculated PME. A batch of dog chews was manufactured and the product rendered micronutrient complete to allow solus feeding. Following a 3 d standard diet pre-feed phase, the test product was fed solus to a panel of seven adult dogs for a period of 8 d. Dietary intake was recorded daily and faecal matter collected for the last 5 d. Test product and pooled faecal samples were analysed for proximate nutrients, and digestibility coefficients were calculated as the difference in intake and faecal excretion (7–11 d). Digestible energy was converted to ME by correcting for energy losses in urine. PME was calculated using proximate analysis and modified Atwater factors according to National Research Council 2006. The results showed close agreement between actual ME (1272 (sd 12·1) kJ/100 g) and calculated PME (1268 (sd 12·6) kJ/100 g), indicating transferability of the NRC 2006 PME equations to the dog chew tested here.

Dog care and treat (C&T) products are often used by dog owners as a means of bonding, for training reinforcement and to deliver functional benefits to their pet. The dog chew studied here is a twin-screw extruded chew with proven efficacy in reducing the build up of dental calculus in dogs^(^^1^^)^. This product must be fed daily to provide the documented benefits so it is important to know the energy density of the product to understand its contribution to the recommended daily energy intake. This knowledge is applied to feeding guides, which help owners balance their pets’ energy consumption and avoid inadequate nutrient intake when considering nutritionally incomplete C&T products. The most accurate way of measuring the energy content of pet foods is via animal feeding studies. Due to the labour intensity and cost of this method, predictive equations are routinely used. The widely used National Research Council (NRC) (2006) predicted metabolisable energy (PME) equations have been derived from main meal feeding study data^(^^2^^)^. Main meal pet food is produced within a processing environment which is substantially different from that used to produce many C&T products. Dry main meal pet food is generally manufactured using single-screw extrusion at a moisture content of about 30 %, with a subsequent drying step, whilst wet main meal pet food is generally manufactured at a moisture content of about 80 %. The chew in the present study was processed by twin-screw extrusion at a moisture content close to its final moisture content (about 15 %). Given this, it is unclear whether the NRC (2006) PME equations are applicable to the dog chew. The aim of the present study was to directly measure the metabolisable energy (ME) content of this C&T product in a panel of dogs and to compare with calculated PME.

## Experimental methods

A panel of seven healthy adult neutered dogs of various breeds was studied. Within the panel were three males and four females, ranging in age from 1 year and 6 months to 4 years and 3 months, body weights ranging from 9·1 to 30·7 kg (mean 18·9 kg). The study took place at the Verden Pet Centre (Mars Petcare) and all methods were conducted in accordance with paragraph 11 of the Animal Protection Law as approved by the Veterinary Inspection Office, Germany. Dogs were housed in pairs throughout the study in kennels equipped with indoor and outdoor runs. Each dog received a daily socialisation period of 40 min and had *ad libitum* access to water throughout the 11 d main study.

To investigate the energy density of the dog chew (PEDIGREE^®^ Dentastix™; Mars Petcare), the product was rendered nutritionally adequate for solus feeding (i.e. the test product represented the only nutrient source) by the addition of vitamins and minerals. The tartar sequestrant sodium tripolyphosphate (STPP) was removed from the product to ensure appropriate P levels. A single batch of this adapted product (‘test product’) was manufactured using a twin-screw extruder and cut into kibbles.

A preliminary study was conducted to assess faeces quality and general health of the dogs in association with solus feeding of the test product. The study consisted of a 2 d pre-feed of standard diet (PEDIGREE^®^ Adult chunk in loaf) followed by a 3 d period of feeding the test product solus. Faeces were scored throughout the 5 d study until 12.00 hours on day 6, using a grading system of 1 to 5 according to the method of Moxham^(^^3^^)^.

The main study comprised a 3 d pre-feed phase where dogs received standard diet as described previously, followed by an 8 d phase of feeding the test product solus. Dogs received the test product as two meals per d, to provide their individual daily energy requirement according to the following equation: 95 kcal/kg body weight^0·75^ per d (397 kJ/kg body weight^0·75^ per d). During the adaptation phase (4–6 d), no samples of faeces or diet were collected to allow for complete transition of test product through the digestive tract. During the collection phase (7–11 d), faeces were collected daily, pooled for each dog and stored at −20°C prior to analysis. Food intakes and refusals were recorded daily.

Faeces and test diet samples were analysed by a commercial laboratory (Eurofins Institute Dr Appelt Southwest GmbH) for proximate nutrient content (moisture, protein, fat, fibre and inorganic matter). Prior to analysis, diet and faeces were ground through a 1 mm screen and homogenised. Protein was analysed according to the Kjeldahl method. Fat and fibre contents were determined according to § 64 LFGB-Methode F 0003. (This reference corresponds to EG 152/2009 and VDLUFA Bd. III 4.1.1.) Ash content was determined via treatment in a muffle furnace at 550°C for 5 h. N-free extract (NFE) was calculated as 100 minus the sum of percentages of moisture, protein, fat, ash and fibre. Bomb calorimetry was used to determine gross energy (GE).

### Calculations and statistical analysis

PME was calculated using proximate analysis in combination with modified Atwater factors, using the following equations:
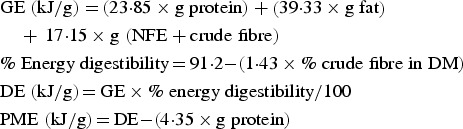


Digestibility coefficients for DM, energy, protein, fat, ash, organic matter and NFE were determined for each dog as apparent digestibility ((consumed − faecal)/consumed). From these, means and standard deviations were calculated using Microsoft^®^ Excel^®^ 2013 (version 15.0.4911.1000). ME was calculated by applying a correction factor of 1·25 kcal/g (5·23 kJ/g) digestible crude protein to the calculated digestible energy (DE) to account for energy lost in urine^(^^4^^)^. Energy release from hindgut fermentation was not taken into account as this is considered to be negligible in dogs^(^^5^^)^.

## Results

Faeces quality during the preliminary study was excellent with 100 % of faeces voided being acceptable throughout.

During the main study, all dogs remained healthy throughout and consumed all food offered. Individual mean daily faeces voided ranged from 68 to 161 g. Body weights remained stable throughout the experimental period within 10 %. Proximate nutrient analysis of the test product is shown in Table 1.

**Table 1.** Analysed proximate nutrient content of the test product

Nutrient (g/100 g)	Test product*
Moisture	15·25
Protein	7·29
Fat	1·50
Fibre	0·20
Ash/inorganic matter	6·07
NFE	69·70

NFE, N-free extract.

* Values shown are means of analyses run in duplicate and are shown on an ‘as is’ basis.

Actual proximate values from each analysis set was used to calculate PME. Calculated GE, DE and energy digestibility are shown in Table 2. The resulting calculated PME was determined as 1267 (sd 12·6) kJ/100 g. When compared with the analysed value, GE was about 3 % lower when using modified Atwater factors. Calculated energy digestibility was overestimated by about 4 % relative to the analysed value.

**Table 2.** Calculated and analysed energy values of the test product

Item	Calculated*	sd	Analysed†	sd
GE (kJ/100 g)	1431·8		1479·0	
DE (kJ/100 g)	1300·8		1292·4	
Energy digestibility (%)	90·9		87·4	
ME (kJ/100 g)	1269·4	12·6	1274·0	12·1

GE, gross energy; DE, digestible energy; ME, metabolisable energy.

* Values shown are means of two calculations, using duplicated proximate analysis of sample.

† GE was determined by bomb calorimetry. DE, energy digestibility and ME were derived from means of values from seven dogs.

## Discussion

The results of the present study indicate that the main meal-derived PME equation is transferable to the twin-screw extruded C&T test product investigated here, based on the close agreement between measured ME and calculated PME.

As the pet food industry adapts to the changing needs and limitations of the world, recipes and processing methods have altered. As a consequence, the accuracy of generalised predictive equations for ME is routinely challenged and a number of studies have previously investigated the correlation between experimental and estimated ME in main meal dog foods^(^^6^^–^^9^^)^. However, to our knowledge, this is the first study to directly measure the ME value of a twin-screw extruded C&T product. This is likely to be due to the fact that many C&T products are not nutritionally complete, which hampers the design of solus feeding studies. Indeed, the majority of C&T products are designed to be fed as a small proportion of the total daily energy allowance whilst the main meal diet is responsible for providing all of the required nutrients.

A retrospective review of digestibility studies in dogs and cats concluded that the error associated with energy digestibility was largely responsible for the variance between PME and actual ME^(^^6^^)^. In the present study, the calculated energy digestibility of the test product was 4 % higher than the analysed value. Overestimation of energy digestibility is likely to cause an underestimation of PME using the modified Atwater factors and NRC equation. However, the calculated GE value in the present study was underestimated by 3 % when taking into account the analysed value obtained by bomb calorimetry. The modified Atwater factors assume constant macronutrient digestibility coefficients and have been challenged for being a source of error when calculating PME. The overestimated energy digestibility had a ‘normalising’ effect on the underestimated GE and the final values for analysed and calculated ME were very similar.

It may be argued that the alterations to the test product to make suitable for solus feeding could have influenced the outcome. However, the added micronutrients and the removed sodium tripolyphosphate (STPP) would have had no effect on energy content and, on this basis, it is assumed that the test product used here is representative of the marketed product.

In conclusion, understanding the energy content of the test product enables the development of accurate feeding guides that allow this functional product to be fed daily in balance with the total recommended daily energy requirement. Assuming compliance by owners, accurate feeding guides help to avoid overfeeding and the inevitable consequences of obesity. Although further work is required to assess repeatability and applicability across other extruded products, the present study indicates that the NRC 2006 method for predicting ME is adequate for the product tested here.
